# Association of cardiorespiratory fitness level with vascular function and subclinical atherosclerosis in the elderly

**DOI:** 10.1007/s00421-023-05375-1

**Published:** 2023-12-22

**Authors:** Vincent Größer, Christopher Weyh, Tim Böttrich, Torsten Frech, Svenja Nolte, Natascha Sommer, Magdalena Huber, Klaus Eder, Oliver Dörr, Sophie Hoelscher, Rebecca Weber, Ebru Akdogan, Holger Nef, Astrid Most, Christian W. Hamm, Karsten Krüger, Pascal Bauer

**Affiliations:** 1 Department of Cardiology and Angiology, Justus- Liebig-University Giessen, 35390 Giessen, Germany; 2 Department of Exercise Physiology and Sports Therapy, Institute of Sports Science, Justus-Liebig-University, Giessen, Germany; 3 Department of Internal Medicine, Member of the German Center for Lung Research, Universities of Giessen and Marburg Lung Center, Justus-Liebig-University Giessen, Giessen, Germany; 4 Institute for Systems Immunology, Center for Tumor und Immunology, Marburg, Germany; 5 Institute of Animal Nutrition and Nutrition Physiology, Justus-Liebig-University, Giessen, Germany; 6 Department of Cardiology, Kerckhoff Clinic GmbH, Bad Nauheim, Germany

**Keywords:** Subclinical atherosclerosis, Cardiorespiratory fitness, Elderly athletes, Pulse wave analysis, Vascular function, Cardiovascular risk factors

## Abstract

**Purpose:**

Physical exercise is crucial for healthy aging and plays a decisive role in the prevention of atherosclerotic cardiovascular disease (ASCVD). A higher level of cardiorespiratory fitness (CRF) in the elderly is associated with lower cardiovascular and all-cause mortality. This study investigated the association of CRF level with vascular function and cardiovascular risk factors in the elderly.

**Methods:**

We examined 79 apparently healthy and physically active subjects aged > 55 years (64 ± 4 years). Cardiovascular functional parameters assessed included brachial and central blood pressure (BP), pulse wave velocity (PWV), augmentation index (Aix), and ankle-brachial index. Sonography of the common carotid artery was performed. CRF level was determined by a cardiopulmonary exercise test, and everyday activity was quantified with an accelerometer.

**Results:**

All participants had a higher CRF level than the reported age-specific normative values. Twenty-nine subjects had subclinical atherosclerosis of the common carotid artery. Compared with participants without atherosclerosis, they were older (*p* = 0.007), displayed higher brachial systolic BP (*p* = 0.006), and higher central systolic BP (*p* = 0.014). Lower brachial (*p* = 0.036) and central (*p* = 0.003) systolic BP, lower PWV (*p* = 0.004), lower Aix (*p* < 0.001), lower body fat percentage (< 0.001), and lower LDL cholesterol (*p* = 0.005) were associated with a higher CRF level.

**Conclusions:**

In this cohort of healthy and physically active individuals, subjects with subclinical atherosclerosis displayed higher systolic brachial and central BP. A higher CRF level was associated with enhanced vascular function, consistent with an influence of CRF on both BP and vascular function in the elderly.

## Introduction

Cardiovascular disease (CVD) is one of the most important causes of death worldwide (Vaduganathan et al. 2022). According to the World Health Organization (WHO), 17.9 million people died of CVD in 2019, equivalent to 32% of all global deaths. Atherosclerosis is the pathology underlying most forms of CVD, including myocardial infarction, heart failure, claudication, and stroke (Lacy et al. 2021; Li et al. 2023; Libby et al. 2019). Atherosclerosis is characterized by structural alterations of the vascular wall resulting in the formation of atherosclerotic plaques (Camaré et al. 2017; Libby et al. 2019). Due to the usually long latency period between the occurrence of atherosclerotic lesions and their clinical manifestation, the diagnosis of subclinical atherosclerosis and the modification of risk factors are of particular importance (Ibanez et al. 2021; Sebastian et al. 2023).

The leading risk factor for the development of atherosclerosis is age, with a significantly increased incidence of CVD in the elderly (Rodgers et al. 2019). The cardiovascular system undergoes structural and functional changes with age, including arterial wall thickening, reduced arterial compliance, endothelial dysfunction, and, consequently, increased systolic blood pressure (BP). These changes lead to a higher susceptibility to the development of atherosclerosis (Lakatta & Levy 2003). In addition to age and arterial hypertension (Jeemon et al. 2021), hyperlipidemia, diabetes, smoking, obesity (Blüher et al. 2019), chronic inflammation, and physical inactivity are known major risk factors associated with an increased risk for the development of atherosclerosis (Libby et al. 2019). Most of these factors can be influenced, at least partly, by changes in lifestyle and are, therefore, of great interest in the context of prevention (Li et al. 2023; Blüher et al. 2019).

There is overwhelming evidence for the health benefits of physical exercise, especially in the context of CVD prevention. Physical activity counteracts age-related changes in the cardiovascular system (Jakovljevic 2018) and positively influences most of the traditional risk factors, such as BP (Liu et al. 2017), insulin sensitivity (Newsom et al. 2013), and cholesterol levels (Mann et al. 2014). Regular exercise lowers all-cause and cardiovascular mortality (Kim et al. 2022). At the same time, physical inactivity favors the development of CVD, diabetes, and some forms of cancer (Harridge & Lazarus 2017). Even moderate exercise such as regular walking significantly reduces the risk of coronary heart disease (Zheng et al. 2009). However, only one in four adults worldwide meets the recommended 150–300 min of moderate physical exercise per week.

The effects of physical activity on vascular function and BP has been particularly well-studied. There is a transient increase in mean arterial pressure during exercise, but in the long-term BP at rest is reduced. The effects are mainly due to reduced peripheral resistance and improved endothelial function as a result of increased NO secretion (Nystoriak & Bhatnagar 2018). Several meta-analyses showed that regular physical exercise leads to a reduction in BP and lowers cardiovascular mortality risk (Pescatello et al. 2019). The BP-lowering effect of exercise is, in some cases, almost comparable to that of drug-based antihypertensive monotherapy (Noone et al. 2020).

In addition, cardiorespiratory fitness (CRF), quantified as maximum oxygen uptake (VO_2_ max), is considered to be an independent risk factor for mortality and an independent predictor of life expectancy in both healthy and CVD patients (Harber et al. 2017). VO_2_ max varies in individuals from values above 90 ml/kg/min in elite endurance athletes to values far below 20 ml/kg/min in the elderly or people with cardiovascular and lung disease (Burtscher 2013). With age, VO_2_ max decreases by about 10% per decade (Pimentel et al. 2003). Despite this age-related decrease and a genetic predisposition, VO_2_ max can be positively influenced by physical exercise throughout a lifetime (Bouchard et al. 2011).

A higher level of CRF is associated with longer disability-free life years (healthy life years) (Strasser & Burtscher 2018) and with lower cardiovascular risk (Chu et al. 2020). Hence, the American Heart Association now recommends assessing CRF in clinical practice (Ross et al. 2016).

Despite the protective effects of a high fitness level on cardiovascular risk, the development of atherosclerosis is multi-factorial and not one isolated risk factor may explain the development in the specific individual. A healthy and active lifestyle can probably reduce the risk of developing atherosclerosis, but cannot eliminate it.

In the general population of Germany, the prevalence of subclinical atherosclerosis in the carotid artery was found to be 43.2% in men and 30.7% in women at the age of 59.5 years (Erbel et al. 2008). A similar prevalence was also found in another cohort (Fernández-Friera et al. 2015).

Hence, we speculated to find individuals with subclinical atherosclerosis even in our cohort of healthy and physically fit elderly individuals free of medication and chronic diseases and a low cardiovascular risk.

The aim of the study was to examine which risk factors were associated with subclinical atherosclerosis in this setting. The cohort of elderly participants with high-fitness levels, and low cardiovascular risk offers a unique opportunity to examine these associations.

We, therefore, determined the subclinical atherosclerotic burden in this apparently healthy cohort (Sebastian et al. 2023) and compared participants presenting with subclinical atherosclerosis with those without. We hypothesized that participants with atherosclerosis would display worse vascular function, higher BP, and lower exercise performance compared with those without subclinical atherosclerosis.

## Methods

### Study design

All participants of the study underwent pre-screening to identify inclusion and exclusion criteria. This screening examination was performed at the University Hospital of Giessen and a sports medical examination was conducted at the Department of Exercise Physiology and Sports Therapy. The cardiovascular screening included sonography of the carotid artery and non-invasive measurement of central and peripheral BP. VO_2_ max was determined with a cardiopulmonary performance test. In addition, venous blood samples were collected for subsequent analysis. Age, height, weight, and body mass index (BMI) were measured. Body fat percentage was assessed with bioelectrical impedance analysis. In addition, daily activity was quantified with step counters (Fitbit Charge 2, Fitbit International Limited, Dublin, Ireland) that were worn for 7 consecutive days.

All participants provided their written informed consent. The ethics committee of the University of Giessen approved the study protocol. The study was performed in accordance with the ethical standards laid down in the Declaration of Helsinki and its later amendments.

### Study population

A total of 79 participants were included in the study. All participants were older than 55 years, free of disease, free of any medication, and were non-smokers. All participants were subjected to a physical examination, cardiovascular screening, and a sports medical examination.

### Carotid sonography

The examinations were performed using a Philips cx50 device (Philips, Eindhoven, the Netherlands) with linear transducer operating at a frequency of 3–12 MHz. with participants placed in a supine position and pillow under the neck. The examination of the carotid arteries was undertaken bilaterally in cross- and longitudinal section from the exit of the subclavian artery to the bifurcation of the internal and external carotid artery in the B-mode to detect and quantify atherosclerotic wall changes. Multiple images of both the left and right common carotid arteries were obtained. Phillips QLAB (Philips Eindhoven, The Netherlands), a semi-automated edge-detection software, was utilized to measure the intima-media thickness over the distal wall of a common carotid artery segment that lies within 1–1,5 cm of the carotid bifurcation in the longitudinal view. The detection box analyses 10 mm length and was placed over the far wall of the common carotid artery segment located within 1–1,5 cm of the carotid bulb. Intima-media edges were determined and IMT during end-diastole were calculated and expressed in millimeters. Success rate, expressed in percentage, is a measure of the edge-detection quality within the detection box; only IMT frames with ≥ 95% success rate were used for analyses. Three frames on each side were analyzed offline by a single-blinded reader and the mean values of all six IMT measurements for each patient were recorded and used for analyses. In case of the detection of subclinical atherosclerosis in the common carotid arteries, no measurements of IMT were undertaken.

### Non‐invasive assessment of peripheral and central blood pressure and pulse pressure waveforms

We used the non-invasive vascassist2® device (isymed GmbH, Butzbach, Germany) to acquire pulse pressure waveforms by means of oscillometry. The device uses a validated model of the arterial tree that consists of 721 electronic circuits representing all central and peripheral arterial sections. By modulating the circuits capacitance, resistance, inductance, and voltage, the system replicates an individual’s acquired pulse pressure waves. The vascassist2® system is currently unique in the use of genetic algorithms to optimize the fidelity of the pulse pressure wave replication. Fidelity replications of 99.6% or above were included in the analysis. The non-invasive vascular evaluation was carried out for all participants after a 15-min rest period. Measurements were performed in a supine position using four conventional cuffs adapted to the upper arm and forearm circumferences of the participants. Radial and brachial pulse pressure waves were acquired on both arms with step-by-step deflation of the cuffs. The measurements took place in a room with a comfortable and stable temperature of 22 °C and a lack of external stress influences. Participants were advised not to move during the acquisition of pulse pressure waves. Two brachial and three radial measurements were performed to guarantee stable and valid results with a break of 30 s between each measurement phase. The total duration of the examination was 15 min. The acquired pulse pressure waves were then analyzed with a validated electronic model of the arterial tree to assess vascular functional parameters. Brachial and radial systolic (SBP) and diastolic (DBP) BP, central systolic and diastolic BP (CBP), pulse wave velocity (PWV), augmentation index (Aix), augmentation index at a heart rate of 75 bpm (Aix@75), resistance index (R), total vascular resistance, and ejection duration were calculated. CBP was determined using a validated transfer function that was based on the peripheral arterial waveform. Calculation of Aix@75 was also based on the pulse waveform. In addition, the vascassist2® device calculates a so-called “vascular age” using the resistance index, arterial stiffness (measured as central pulse wave velocity), and the calendar age. In the first step, a "resistance age" is calculated from the resistance index (dmult). In the second step, a “PWV age” is calculated from the PWV. In the third step, PWV age, resistance age, and calendar age are averaged to give the vascular age. The calculation method is shown below:

- Resistance age = [ln (-0.0714286 + (0.0178571 * dmult)) – ln 0.09298] / 0.02532
- PWV age = [ln aoPWV – ln 5.05734] / 0.00845
- Vascular age = (resistance age + PWV age + calendar age) / 3

### Quantification of daily activity by analyzing step counters

The participants wore a pedometer (Fitbit Charge 2, Fitbit International Limited, Dublin, Irland) for seven consecutive days, and the step count of each day was calculated separately. The step count of an average week is considered to be representative of typical everyday activity, and the mean number of steps taken per day over 7 days was used for statistical analysis.

### Cardiopulmonary exercise test

The participants were subjected to a ramp test on an electromagnetically braked bicycle ergometer (Excalibur SportR, Lode B.V., Groningen, Netherlands) and maximum oxygen uptake (VO_2_ max) or VO_2_ peak were determined. Two different ramp protocols were used. Briefly, the exercise test started with a 3-min warm-up period without resistance. The exact ramp protocol was selected depending on the training or fitness status of the participant, with the aim of reaching the maximum load after 15 min. Before the test, all participants were asked about their sports history. Participants with no systematic endurance training or competition experience completed a 2-way ramp: 3 min without load followed by a beginning load of 50 W, which was increased every 3 min by 25 W. From 100 W the increase was every 2 min by 25 W. Trained subjects started at 50 W after the 3-min warm-up period without load and increased by 50 W every 3 min. The test was performed until complete exhaustion.

Any one of the following criteria was used to verify exhaustion:(a) request of the participant due to extreme tiredness and/or perception of intense dyspnea; (b) reaching 85% of the maximum heart rate (HR) predicted by age (HR max); (c) attaining a peak respiratory exchange ratio RER > 1.1; (d) reaching the VO_2_ plateau even with increasing workload. Ventilatory and metabolic parameters were collected by respiration using Metalyzer 3-B (Cortex, Germany) and were analyzed. The average of the last 30 s of the test was used to determine VO_2_ peak.

### Blood sampling and testing

Venous fasting blood samples (80 ml) were taken from each participant between the hours of 08:00–10:00 for further analysis. Fasting concentrations of glucose, HbA1c, total cholesterol, low-density lipoprotein (LDL), high-density lipoprotein (HDL), and triglycerides were measured directly by standard clinical laboratory methods by the laboratory of Synlab in Bad Nauheim (Germany).

### Statistical analysis

Descriptive analyses were carried out on all study variables for the entire cohort. Data are presented as means ± standard deviation (SD). Comparisons between the group without and the group with subclinical atherosclerosis were performed using *t* tests on independent samples. The Levene test was used to determine the homogeneity of the variances. For homogeneous variants, the Student's t-test was performed. For a skewed distribution, the Welch test was used. The Shapiro–Wilk test was used to determine whether the samples were normally distributed. For non-normally distributed samples, the Mann–Whitney U test was used as a non-parametric method. Bivariate relations were analyzed using Pearson’s product–moment correlation coefficient. Multivariable stepwise regression analyses were carried out to explore possible linear associations. Statistical significance was set at *p* < 0.05 (two-tailed) for all measurements. All statistical analyses were performed using JASP for IOS, version 0.9.1 (JASP, Amsterdam, Netherlands).

## Results

### Cohort characteristics

The average age of the participants was 64 ± 4 years, ranging from 56 to 75 years. The cohort comprised 49 male participants and 30 females. Twenty-nine (36.7%) of all participants examined showed atherosclerotic changes of the carotid artery. Based on these results, the participants were divided into two groups, one with and one without subclinical atherosclerosis.

Average values for BMI, body fat, BP, glucose metabolism, cholesterol levels, VO_2_ peak and daily activity are listed in Table 1. Based on the BMI, 26 participants were overweight (25 to 29.9 kg/m^2^) and 4 had first-degree obesity (30 to 34.9 kg/m^2^), while the majority of the participants (62%) had a normal BMI. According to the definition of the European Society of Cardiology (Williams et al. 2018), 48 participants had normal or optimal BP, 15 had high-normal BP (systolic: 130–139 mmHg and/or diastolic: 85–89 mmHg), 6 were classified as having grade 1 hypertension (systolic: 140–159 mmHg and/or diastolic: 90–99 mmHg), and 1 participant had grade 2 hypertension (systolic: 160–179 mmHg and/or diastolic: 100–109 mmHg). None of the participants had an HbA1c level above 6.5%, which would correspond to the WHO criterion for diabetes.

**Table 1:** Characteristics of all 79 participants

	Mean	SD
Age (years)	63.7	3.7
BMI (kg/m^2^)	24.9	3.1
Body fat (%)	27.2	6.5
SBP (mmHg)	129	13
DBP (mmHg)	76	10
Fasting glucose (mg/dl)	99	8.5
HbA1c (%)	5.5	0.3
Cholesterol (mg/dl)	217	33
LDL-Cholesterol (mg/dl)	147	34
VO_2_ peak (ml/min/kg)	30.0	7.3
Steps/day	11,500	4000

Only 14 test subjects were found to have low or medium cardiovascular risk according to the European Society of Cardiology target values ​​for LDL cholesterol < 115 mg/dl (Mach et al. 2019)**.** Fifty-seven participants had LDL values between 115 and 200 mg/dl and 4 were above 200 mg/dl. The mean VO_2_ peak of all participants was 30.0 ± 7.3 ml/min/kg (Table 1). The participants walked 11,500 ± 4000 steps per day.

### Characteristics according to the presence of subclinical atherosclerosis

**Fig. 1:**
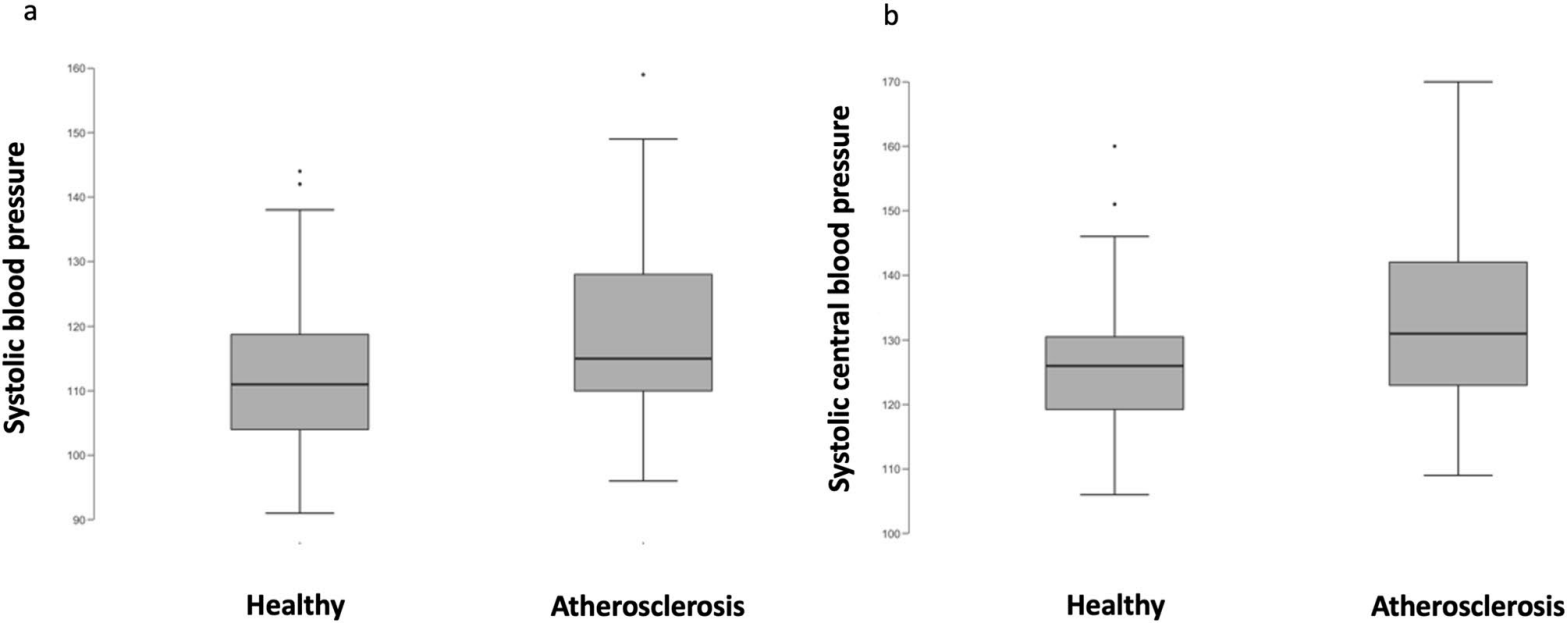
Differences in systolic blood pressure (SBP) according to the presence of atherosclerosis. Differences in systolic central blood pressure (sys CBP) according to the presence of atherosclerosis

Participants with subclinical atherosclerosis were significantly older than participants without (*p* = 0.007) (Table 2). No differences in BMI and body fat were detected between the groups. Participants with subclinical atherosclerosis had on average 8 mmHg higher brachial SBP (*p* = 0.006), higher mean BP (*p* = 0.028), higher systolic CBP (*p* = 0.014) and mean CBP (*p* = 0.028) values compared to those without atherosclerosis (Fig. 1). Furthermore, the calculated mean vascular age was 4.3 years higher in participants with subclinical atherosclerosis (*p* = 0.008). No statistically significant differences between the groups were detected in the other vascular parameters, and there were also no differences in cholesterol levels, fasting glucose, or HBA1c. (Table 2).

**Table 2:** Comparison of anthropometric data and cardiovascular parameters according to the presence of subclinical atherosclerosis

	Healthy (n = 49)		Atherosclerosis (n = 29)		p value
	Mean	SD	Mean	SD	
Age (years)	63	3.5	65	3.6	0.007*
BMI (kg/m^2^)	25.1	3.0	24.8	3.2	0.653
Body fat (%)	27.9	6.6	26.0	6.3	0.885
SBP (mmHg)	126	11	134	15	0.006*
DBP (mmHg)	74	10	77	10	0.143
MAP (mmHg)	92	9	96	10	0.028*
CBP systolic (mmHg)	112	12	119	16	0.014*
CBP diastolic (mmHg)	75	11	77	11	0.148
MAP central (mmHg)	93	10	98	11	0.028*
PWV (m/s)	7.8	0.8	8.0	1.2	0.414
AIX @75 bpm	8.6	13.3	11.4	12.2	0.173
ABI (mean)	1.24	0.06	1.20	0.15	0.092
Vascular age (years)	54	7	59	8	0.008*
Fasting glucose (mg/dl)	99	8	100	9	0.422
HbA1c (%)	5.5	0.3	5.5	0.4	0.269
Cholesterol (mg/dl)	221	34	211	31	0.885
Triglycerides (mg/dl)	113	88	89	36	0.869
LDL (mg/dl)	148	34	145	36	0.671
HDL (mg/dl)	62	16	62	14	0.481
LDL/HDL ratio	2.6	0.9	2.5	1.0	0.686
VO_2_ peak (ml/min/kg)	29.9	7.3	30.5	7.3	0.657
Maximum power (Watts/kg)	2.6	0.6	2.6	0.6	0.471
Steps/day	11,800	4100	10,900	3700	0.182

*Statistically significant difference (*p* < .05)

No statistically significant differences between the groups were detected in VO_2_ peak, relative peak power and daily activity (Table 2).

### Correlation between fitness parameters and vascular function and cardiovascular risk factors

**Fig. 2:**
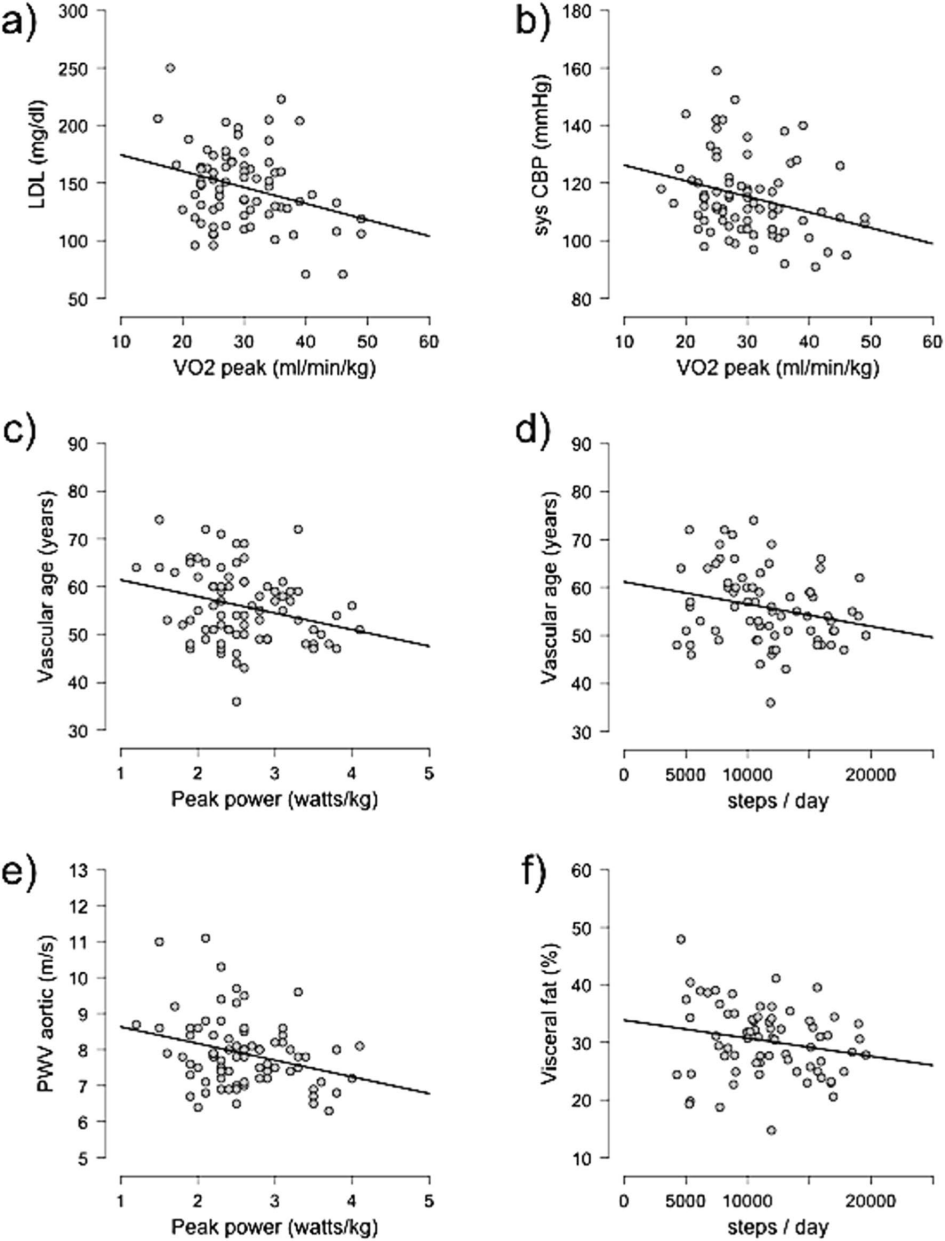
Correlations between fitness markers and several cardiovascular functional marker: **a** Correlation between VO_2_ peak and LDL cholesterol levels. **b** Correlation between VO_2_ peak and systolic CBP. **c** Correlation between peak power and vascular age. **d** Correlation between the steps walked per day and vascular age. **e **Correlation between peak power and aortic PWV. **f** Correlation between the steps walked per day and percentage of visceral fat

Correlations between the parameters of performance, daily activity, and cardiovascular risk factors and parameters of vascular function were assessed. The results are provided in Table 3 and depicted in Fig. 2.

**Table 3:** Pearson correlations between cardiorespiratory fitness markers and serveral cardiovascular functional markers

	VO_2_ peak		Watt/kg		Steps/day	
	r	p value	r	p value	r	p value
SBP (mmHg)	-0.169	0.069	-0.203	0.036*	-0.043	0.361
MAP (mmHg)	0.038	0.631	-0.068	0.275	0.000	0.501
CBP sys. (mmHg)	-0.282	0.006*	-0.301	0.003*	-0.017	0.445
Central MAP (mmHg)	-0.161	0.078	-0.181	0.055	-0.009	0.470
PWV (m/s)	-0.264	0.009*	-0.292	0.004*	-0.202	0.044*
Augmentation index @75 (%)	-0.433	< 0.001*	-0.407	< 0.001*	-0.061	0.305
Intima media thickness (mm)	0.014	0.537	0.017	0.453	0.053	0.632
Vascular age (years)	-0.200	0.038*	-0.276	0.007*	-0.236	0.023*
BMI (kg/m^2^)	-0.304	0.004*	-0.394	< 0.001*	-0.124	0.153
Body fat (%)	-0.713	< 0.001*	-0.708	< 0.001*	-0.181	0.064
Visceral fat (%)	-0.629	< 0.001*	-0.637	< 0.001*	-0.206	0.043*
Cholesterol (mg/dl)	-0.224	0.026*	-0.254	0.014*	-0.127	0.150
LDL (mg/dl)	-0.296	0.005*	-0.294	0.005*	-0.046	0.355
HDL (mg/dl)	0.118	0.157	0.119	0.155	-0.022	0.570
LDL/HDL	-0.225	0.026*	-0.231	0.023*	0.020	0.564
Triglycerides (mg/dl)	-0.099	0.200	-0.140	0.117	-0.127	0.150

*Statistically significant correlation (*p* < .05)

VO_2_ peak was negatively correlated with systolic CBP (*r* = − 0.282, *p* = 0.006), PWV (*r* = − 0.264, *p* = 0.009), Aix@75 (*r* = − 0.433, *p* < 0.001), vascular age (*r* = − 0.200, *p* = 0.038), BMI (*r* = − 0.299, *p* = 0.008), total body fat percentage (*r* = − 0.713, *p* < 0.001), visceral fat percentage (*r* = − 0.629, *p* < 0.001), total cholesterol (*r* = − 0.224, *p* = 0.026), LDL cholesterol (*r* = − 0.296, *p* = 0.005), and the LDL/HDL ratio (*r* = − 0.225, *p* = 0.026). There was also a negative correlation between peak performance in W/kg and brachial SBP (r = − 0.203, *p* = 0.036), systolic CBP (r = − 0.301, *p* = 0.003), PWV (r = − 0,292, *p* = 0.004), Aix@75 (r = − 0.407, *p* < 0.001), vascular age (r = − 0.276, *p* = 0.007), BMI (r = − 0.377, *p* < 0.001), total body fat percentage (r = − 0.708, *p* < 0.001), visceral fat percentage (r = − 0.637, *p* < 0.001), total cholesterol (r = − 0.254, *p* = 0.014), LDL cholesterol (r =—0.294, *p* = 0.005), and LDL/HDL ratio (r = − 0.231, *p* = 0.023). The number of steps walked was negatively correlated with PWV (r = − 2.202, *p* = 0.044), vascular age (r = − 0.236, *p* = 0.023) and the percentage of visceral fat (r = − 0.206, *p* = 0.043).

Furthermore, linear regression analyses revealed that a higher VO_2_ peak is a significant predictor of lower systolic CBP (*R*^2^ = 0.079, *p* = 0.012), lower PWV (*R*^2^ = 0.070, *p* = 0.019), lower Aix@75 (*R*^2^ = 0.187, *p* < 0.001), lower BMI (*R*^2^ = 0,092, *p* = 0,007), and lower total (*R*^2^ = 0.508, *p* < 0.001) and visceral (*R*^2^ = 0.396, *p* < 0.001) body fat percentage as well as LDL-cholesterol (*R*^2^ = 0.088, *p* = 0.010).

Peak performance in watts per kilogram of body weight was found to be a significant predictor of lower systolic CBP (*R*^2^ = 0.091, *p* = 0.007), lower PWV (*R*^2^ = 0.086, *p* = 0.009), lower Aix@75 (*R*^2^ = 0.166, *p* < 0.001), lower vascular age (*R*^2^ = 0.076, *p* = 0.014), lower BMI (*R*^2^ = 0.154, *p* < 0.001), and lower total (*R*^2^ = 0.502, *p* < 0.001) and visceral (*R*^2^ = 0.405, *p* < 0.001) body fat percentage as well as lower total cholesterol (*R*^2^ = 0.064, *p* = 0.028), LDL-cholesterol (*R*^2^ = 0.087, *p* = 0.010), and LDL/HDL ratio (*R*^2^ = 0.053, *p* = 0.046).

Walking more steps per day was shown to be a predictor of a lower vascular age (*R*^2^ = 0.056, *p* = 0.046).

## Discussion

The prevention of atherosclerosis is a crucial task for disability-free and healthy ageing. As physical activity is the major tool to modify cardiovascular risk factors, prevent atherosclerosis, and achieve better CRF, we aimed to evaluate the association of CRF with cardiovascular risk factors and subclinical atherosclerosis in an apparently healthy elderly population free of medication and disease.

Unlike previous studies, the present investigation assessed both cardiovascular, cardiorespiratory status and screened for subclinical atherosclerosis. Hence, in line with recently published data, we found subclinical atherosclerosis in 36.7% of all apparently healthy subjects examined. This prevalence is lower than what has been reported in other European communities (Bergström et al. 2021). Further and in line with the aforementioned, our study cohort was healthier than the general European population, ​​with lower rates of obesity and hypertension (Blüher et al. 2019; Jeemon et al. 2021; Łuniewski et al. 2023; Vaduganathan et al. 2022). Contrary to what would be expected in this age group, none of the participants were diagnosed with diabetes (Gourdy et al. 2022; Tamayo et al. 2014). In addition, we only included participants who were free of medication and nicotine use.

Age is considered to be the strongest risk factor for the development of atherosclerosis (Rodgers et al. 2019), which also applies to our study cohort. Participants with subclinical atherosclerosis were 1.9 years older than participants without. Interestingly, the calculated vascular age was even 4.3 years higher in this group, which indicates that this value may be more meaningful than the calendar age alone.

Although elevated BP values were less common in our collective than in the general population of comparable age, we detected a significant higher SBP (8 mmHg) in the group with subclinical atherosclerosis compared to their peers without atherosclerosis. This observation is consistent with results from previous studies that showed higher cardiovascular risk with increasing BP, even in the normal BP range (Jeemon et al. 2021; Whelton et al. 2020), as defined by current guidelines. Of note, there was no significant difference in DBP between the groups as well as in other established risk factors, such as cholesterol levels, fasting glucose and HbA1c. This highlights the important impact of systolic BP for the development of atherosclerosis, even below the currently recommended threshold of 140 mmHg SBP in the European guidelines (Jeemon et al. 2021).

The participants of our study appeared to have an above-average physical activity level and were well-trained. In comparison with age-and sex- adapted reference values ​​for maximum oxygen uptake from a large study on 10.090 adults (Rapp et al. 2018), our participants were in the upper 20th percentile, highlighting their high fitness level.

However, we could not detect differences in VO_2_ peak and everyday activity between the group with subclinical atherosclerosis compared to the group without atherosclerosis. This might also be explained with the high level of fitness of our participants and the high homogeneity of the performance data. One may speculate that an association between CRF and the development of atherosclerosis could be observed in the general population, where the prevalence of physical inactivity, and therefore, reduced CRF is significantly higher. However, these results emphasize the importance of systolic BP on the development of atherosclerosis even in low-risk individuals with a high cardiorespiratory fitness level.

Nevertheless, there was a strong association between CRF, cardiovascular risk factors and vascular function. A higher CRF level was an independent predictor of a lower systolic CBP, lower PWV and a lower Aix@75, indicating that high levels of CRF lead to improved central hemodynamics and arterial compliance. CBP, PWV and Aix are proven vascular biomarkers that allow an early detection of vascular dysfunction, which also can be observed in the aging process (Battistoni et al. 2020).

Particularly SBP increases with age as a result of reduced arterial compliance of the aorta. We found an association between brachial, central SBP and subclinical atherosclerosis in our study. Former studies suggest that CBP is the more decisive parameter in cardiovascular risk assessment, especially in younger age groups (McEniery et al. 2014). In addition, reduced arterial compliance leads to an increased left ventricular afterload, which promotes left ventricular hypertrophy and diastolic dysfunction (Mottram et al. 2005). The observed association of higher CRF level with reduced CBP, PWV and Aix suggests that regular exercise and high CRF level may serve as a powerful tool to slow down the vascular aging process and, in consequence, reduce cardiovascular risk. The amount of daily activity was also a predictor of the calculated vascular age, which bespeaks this association.

Finally, it should be emphasized that a high CRF level and a higher level of daily activity in our collective were associated with a lower body fat percentage, in particular central and visceral fat mass. Increased body fat and obesity are further established cardiovascular risk factors (Henning 2021). Obesity also leads to an increase in other risk factors, such as BP (Din-Dzietham et al. 2007) and cholesterol levels (Schröder et al. 2003), which shows the multiple cardiovascular health benefits of lowering body fat through physical activity.

CRF is a well-known predictor of cardiovascular events and mortality (Laukkanen et al. 2004; Sui et al. 2007). Hence, integrating CRF, as proven independent risk factor, into established risk scores might have additional benefits for individual cardiovascular risk calculation.

## Limitations

First, this was a cross-sectional analysis, and the subjects were only examined once. Further longitudinal studies are necessary to explore causality. Furthermore, our study cohort was free of medication and displayed an above-average state of health and CRF. The results are, therefore, only partially representative of the general population. Finally, the evaluation of the step counters for assessing daily activity must also be examined critically, since they were only worn for about a week. Thus, it cannot be determined with certainty whether the participants showed a representative amount of daily activity during this week that was comparable to their average amount. The diagnosis of subclinical atherosclerosis and hence the division in the two groups were just based on the results of carotid sonography. No other screening methods such as a coronary calcium scan were performed.

However, the major strength of the study is the homogenous study cohort and the high CRF and physical activity level in elderly individuals without medication. Hence, relevant confounders of assessing associations between CRF, BP and risk factors with atherosclerosis are minimized.

## Conclusion

In our cohort of physically fit individuals with a homogenous high CRF, without cardiovascular disease and free of medication, we detected a significant and independent association of higher brachial and central SBP with the presence of subclinical atherosclerosis, without differences in VO2 peak between the two groups. This highlights the influence of blood pressure, even below the currently defined hypertension criteria in Europe, on subclinical atherosclerosis. Further studies that address the influence of BP levels and the BP burden over time on the development of atherosclerosis are needed in the future.

In addition, we detected a strong association between higher levels of CRF, improved vascular function, and lower rates of major cardiovascular risk factors, such as obesity or elevated cholesterol-levels. Hence, a higher CRF level may counteract vascular aging and lead to lower SBP, thus lowering the risk for developing atherosclerosis.

## Data Availability

The data sets used and/or analyzed during the current study are available from the corresponding author on reasonable request.
